# Heritable heading time variation in wheat lines with the same number of *Ppd-B1* gene copies

**DOI:** 10.1371/journal.pone.0183745

**Published:** 2017-08-28

**Authors:** Zuzana Ivaničová, Miroslav Valárik, Kateřina Pánková, Martina Trávníčková, Jaroslav Doležel, Jan Šafář, Zbyněk Milec

**Affiliations:** 1 Institute of Experimental Botany, Centre of the Region Haná for Biotechnological and Agricultural Research, Šlechtitelů 31, CZ Olomouc, Czech Republic; 2 Crop Research Institute, Drnovská 507, Prague, Czech Republic; 3 Czech University of Life Sciences Prague, Kamýcká 129, Prague 6, Czech Republic; Institute of Genetics and Developmental Biology Chinese Academy of Sciences, CHINA

## Abstract

The ability of plants to identify an optimal flowering time is critical for ensuring the production of viable seeds. The main environmental factors that influence the flowering time include the ambient temperature and day length. In wheat, the ability to assess the day length is controlled by photoperiod (*Ppd)* genes. Due to its allohexaploid nature, bread wheat carries the following three *Ppd-1* genes: *Ppd-A1*, *Ppd-B1* and *Ppd-D1*. While photoperiod (in)sensitivity controlled by *Ppd-A1* and *Ppd-D1* is mainly determined by sequence changes in the promoter region, the impact of the *Ppd-B1* alleles on the heading time has been linked to changes in the copy numbers (and possibly their methylation status) and sequence changes in the promoter region. Here, we report that plants with the same number of *Ppd-B1* copies may have different heading times. Differences were observed among F_7_ lines derived from crossing two spring hexaploid wheat varieties. Several lines carrying three copies of *Ppd-B1* headed 16 days later than other plants in the population with the same number of gene copies. This effect was associated with changes in the gene expression level and methylation of the *Ppd-B1* gene.

## Introduction

The day length plays a crucial role in the plant life cycle and mainly impacts the decision of when to flower. Certain plants require a long day (LD), and certain plants require a short day (SD) before flowering; however, there are also plants that are insensitive to the day length. This behaviour is called a photoperiod response and is genetically controlled. Bread wheat (*Triticum aestivum* L.) is one of the world’s most important staple food sources and provides nutrition to 30% of the human population. Originally, wheat was an LD plant that required at least 14 hours of daylight to flower. When grown under SD conditions, flowering was significantly delayed. However, the introduction of photoperiod-insensitive wheat cultivars facilitated the spreading of wheat cultivation to regions with favourable conditions (e.g., water availability, appropriate temperature) and short daylight.

In wheat, the photoperiod response is controlled by photoperiod (*Ppd*) genes that are located on chromosomes 2A, 2B and 2D. These genes belong to a pseudo-response regulator family [1]. Of these genes, the *Ppd-D1a* allele on chromosome 2D has a major effect on the photoperiod response. The allele causing the photoperiod insensitivity carries a 2,089 bp deletion upstream of the coding region [1]. Another photoperiod-insensitive allele, *Ppd-A1a*, is located on chromosome 2A and differs from the wild type by a 1,085 bp deletion. Interestingly, the deletion occurs at the same gene region as that in the *Ppd-D1a* allele [2]. In contrast, two different mechanisms were identified for the *Ppd-B1* allele. Díaz et al. [3] described the impact of different numbers of *Ppd-B1* copies on the photoperiod response as follows: while the presence of only one copy of *Ppd-B1* causes sensitivity to day length, an increased copy number of *Ppd-B1* is associated with day length insensitivity and, consequently, earlier flowering. The authors also reported different haplotypes of the *Ppd-B1a* allele based on a combination of the presence/absence of amplicons derived from the junctions between the individual *Ppd-B1a* copies. Zhang et al. [4] showed that different haplotypes are responsible for the variation in the heading time, and varieties with *Ppd-B1_Hapl-VI* demonstrated the earliest heading. Furthermore, Nishida et al. [2] described a 308 bp insertion in the promoter region of the winter variety Winter-Abukumawase. This insertion was associated with photoperiod insensitivity, and the allele carrying this mutation was designated *Ppd-B1a*.*1*. The Winter-Abukumawase variety has only one *Ppd-B1* copy (H. Nishida, pers. comm.); therefore, similarly to the *Ppd-D1* allele, the earlier heading appears to be associated with DNA sequence changes in the promoter region.

The copy number variation (CNV) involves deletions or duplications of DNA regions that are typically larger than 1 kb [5,6]. Such DNA rearrangements are often associated with changes in the phenotype. CNVs have been widely studied in humans [7,8] and have been linked with various diseases, such as Parkinson´s disease, which is associated with a triplication of the α-synuclein gene [9]. However, CNVs also played a significant role in the human genome evolution with positive effects on beneficial traits, such as endurance running, which is likely associated with a duplication of the *aquaporin 7* gene [10]. CNVs have also been studied in plants either at the whole genome level [11] or in analyses focusing on the expression of particular genes [3]. Francia et al. [12] reported an increased copy number of genes encoding C-repeat Binding Factor (*HvCBF4* and *HvCBF2*) in barley, which resulted in greater frost tolerance. Another study in barley showed that higher number of *HvFT1* copies (homologue of *FLOWERING LOCUS* T in barley) can also accelerate the flowering time [13].

Variation at the DNA sequence level is not the only factor that influences gene expression. Epigenetic modifications, including DNA methylation and histone modifications, play an important role and may lead to significant changes in the phenotype. In plants, DNA methylation occurs at several particular sites as follows: CG, CHG and CHH, where H stands for A, T or C, while in mammals, mainly the CG site is involved [14,15]. DNA methylation is usually associated with a lower gene expression and gene silencing [16,17]. Nevertheless, Sun et al. [18] observed a higher level of methylation of the *Ppd-B1* gene, which was linked to a higher number of copies of the gene. The authors suggested that the higher level of DNA methylation leads to an increased expression level of *Ppd-B1* and, consequently, an accelerated heading time. This effect might be attributed to reduced binding of a putative repressor to the promoter region, which results in an increased gene expression. An increase in the level of lysine 4 trimethylation in histone H3 (H3K4me3) was implicated in the upregulation of *TaVRN1* and *TaFT1* genes during the vernalization of winter wheat. This upregulated state was maintained throughout plant development and was reset only in the next generation [19]. Similarly, in *Arabidopsis*, the trimethylation of the lysine residue in histone H3 (H3K27me3) leads to a repression of the *FLOWERING LOCUS C* (*FLC*) gene during vernalization and results in the ability to flower [20,21].

In this study, we report differences in heading time among recombinant inbred lines (RIL) F_7_ individuals that carry the same copy number of the *Ppd-B1* allele. These lines were derived by crossing two spring hexaploid wheat varieties with differing *Ppd-B1* copy numbers. Although the parental varieties carry the same set of alleles that control the flowering time (*Vrn-A1a*, *Vrn-B1c*, *vrn-D1*, *VRN-A3*, *vrn-B3*, *Ppd-D1b* and *Ppd-A1b*), several lines with three *Ppd-B1* copies differed significantly in the heading time. The difference was associated with changes in the *Ppd-B1* expression level. The role of the sequence and epigenetic differentiation of the *Ppd-B1* gene and its promoter in the heading time variation was investigated.

## Materials and methods

### Plant material

Seeds of the bread wheat (*Triticum aestivum* L.) cultivars Kaerntner Frueher (KF) and Paragon (P) were obtained from Gene Bank (https://grinczech.vurv.cz/gringlobal) of Crop Research Institute (Prague, Czech Republic). KF is a photoperiod-insensitive spring hexaploid wheat that carries the *Ppd-B1a* allele (three copies of *Ppd-B1*), while P is photoperiod sensitive and carries the *Ppd-B1b* allele (one copy of *Ppd-*B1). The remaining alleles influencing the flowering time are the same in both cultivars (*Vrn-A1a*, *Vrn-B1c*, *vrn-D1*, *vrn-B3*, *Ppd-D1b* and *Ppd-A1b* [22] and *VRN-A3*). The two cultivars were crossed, and an F_2_ population was obtained. Then, RILs were created using the single seed descent method to obtain the F_7_ generation. Plants in the F_7_ population were grown in ten replicates under a controlled regime (20°C/16°C–day/night temperature) in a growth chamber under an artificial LD of 16 hours. Three biological replicates of each line were used to analyse the gene expression and DNA methylation status of *Ppd-B1*.

### QTL analysis

Five seeds of each parental cultivar and all 130 individuals in the F_2_ mapping population were incubated for four days at 6°C to synchronise germination. Seedlings were grown under controlled conditions (20°C/16°C–day/night temperature) under artificial light with a 16-hour LD. The heading date was recorded at the emergence of half of the spike. For the QTL analysis, 90 F_2_ individuals were selected to fit one 96-well PCR plate. DNA was extracted from the leaves using the Invisorb Spin Mini Plant Kit (Stratec Molecular, Berlin, Germany) according to the manufacturer´s instructions. The mixed model QTL analysis [23], genotyping according to the DArT markers [24] and the genetic map construction were carried out by Diversity Arrays Technology (DArT, Canberra, Australia http://www.diversityarrays.com).

### *VRN-A3* sequencing

The *VRN-A3* gene was sequenced to identify variations between the parental cultivars. In total, six primer pairs (Table 1) were used to obtain the complete gene sequence and the 1,750 bp and 143 bp flanking sequences from the 5´ and 3´ end of the gene, respectively.

**Table 1:** Primer pairs used for the sequencing of the *VRN-A3* and *Ppd-B1* genes.

Gene	Name	Sequence (5´- 3´)	Product size (bp)	Reference
*Vrn-A3*	VRN3_F4	CTAAATAGCAAGACGCCACTAT	545	this study
	VRN3_R4	CCTCCTAGAAACTGCCACACA		
	VRN3_F5	TAATGGACCTCCATAGCTAGC	909	this study
	VRN3_R5	GTGGCTTCTAGGCCGTGCC		
	VRN3_F6	CGCAGCTCATACCTTGGACTA	1365	this study
	VRN3_R6	CATCGAGAATCATCTTCCCAC		
	VRN3_F7	ACGTCCACAGAACCAATTCA	604	this study
	VRN3_R7	CATCGAGAATCATCTTCCCAC		
	VRN3_F8	AAATGGCCGGGAGGGAC	552	this study
	VRN3_R8	ACGTAGAGAGTACTACGTGC		
	VRN3_F9	GTTCTGGCAAGCACACGAC	597	[28]
	VRN3_R9a	AATTTGCTGACTTTGCGGGC		
*Ppd-B1*	PromF1	AAGTGTACGTGGTTAACATTAG	623	this study
	PromR1	GGAGTTATCTTAACACTTGC		
	PromF2	GTGCTAAGATAACTTTGTC	546	this study
	PromR2	GAAAGGAAAGAAAGAAGC		
	PromF3	AAATATGCGCTGTATGTC	539	this study
	PromR3	CGTGAACAAGACCAGGACCAG		
	PromF4	GTATAGAGTCAGAAGGAGGGC	522	this study
	PromR4	TGAGTGCCAGATCCAAAAGCTG		
	Ex1_F1	TGCCATATAGATCCTTTCTGATA	448	this study
	Ex1_R1	TTGACACCAACAGCTTCCAG		
	Ppd-B1_101F	CGCCACTGCATGTACCAAGTTA	96	this study
	Ppd-B1_101R	CTGTCAGAACAAGGTCGATGTTG		

A set of six primer pairs was used to sequence the 2,732 bp region, which includes the *VRN-A3* gene (839 bp) and flanking regions (1,750 and 143 bp from the 5´ and 3´ end of the gene). Amplification was performed using a touchdown PCR protocol. For the *Ppd-B1* gene, a set of five primer pairs was used to sequence the promoter region together with a portion of the first intron (from—1,545 bp to + 251 bp).

The *VRN-A3* gene-specific primers were designed using publicly available sequences (GenBank accession nos. DQ890162, EF428115 and EF428119) and chromosome-specific survey sequences of cv. Chinese Spring [25]. All sequences were aligned using Geneious 5.6.4 software [26]. The primer pairs were considered specific when at least one primer contained three or more genome-specific SNPs/indels. The melting temperatures were determined using Primer-BLAST [27]. The PCR reaction mix (15 μl volume) comprised 10 ng of the template DNA, 200 μM of each dNTP, 2 mM of MgCl_2_, 0.2 μM of both primers, 1 × PCR buffer B1 (Solis BioDyne, Tartu, Estonia) and 0.03 U of Hot FirePol *Taq* polymerase (Solis BioDyne, Tartu, Estonia). This mixture was consecutively used for the methylation status determination, cloning and copy number assessment. The DNA amplification was carried out by touchdown PCR as follows: initial denaturation for 13 min at 95°C, followed by 14 cycles at 95°C for 40 s, 65°C for 40 s (increment of -0.7°C/per cycle) and 72°C for 2 min. An additional 19 cycles were performed at 95°C for 40 s, 55°C for 40 s and 72°C for 2 min with a final elongation at 72°C for 10 min. The purification of the product and sequencing were performed as previously described by Ivaničová et al. [28].

### *Ppd-B1* promoter sequencing

The promoter region and a portion of the first exon (from—1545 bp to + 251 bp) of the parental cultivar KF and the F_7_ lines 32_2 and 37_4 were sequenced to determine whether there were sequence variations that could possibly cause the difference in the heading time. The primers (Table 1) were designed based on the Sonora 64 sequence (JF946486.1) using Primer-BLAST [27]. The DNA amplification and purification and the sequencing of the PCR product were performed in same manner as described above for the *VRN-A3* gene.

### RNA extraction and expression analysis

The expression of the *Ppd-B1* gene was assessed in lines of the F_7_ mapping population and the parental cultivars. RNA was extracted from the leaves of 20-day-old seedlings using the RNasy Plant Mini Kit (Qiagen, Hilden, Germany). The leaves were collected from all lines in three biological replicates three hours after dawn when the *Ppd-B1* expression reached the maximum level [3]. DNA was removed during the RNA purification using the RNase-Free DNase Set (Qiagen, Hilden, Germany). cDNA was synthesized using the Transcription High Fidelity cDNA Synthesis Kit (Roche Diagnostics, Mannheim, Germany) according to the manufacturer´s protocol with 2 μg of total RNA and anchored-oligo (dT)_18_ primers.

The gene expression level was determined using reverse transcription-qPCR (RT-qPCR). RT-qPCR was performed using qPCR 2x SYBR Master Mix (Top-Bio, Prague, Czech Republic) on the CFX96TM Real-Time PCR Detection System (Bio-Rad, Hercules—California, USA). The expression level of *Ppd-B1* was standardized against the reference gene *glyceraldehyde-3-phosphate dehydrogenase* (*GAPDH*) according to Sun et al. [18].

The data were analysed using the 2^-ΔΔCq^ method with CFX Manager 3.0 software (Bio-Rad, Hercules—California, USA). Three replicate PCR amplifications were performed for each sample. The transcript level of the target gene *Ppd-B1* in cultivar KF was designated 1.0. The primers for *Ppd-B1* (Ppd-B1_101F: CGCCACTGCATGTACCAAGTTA, Ppd-B1_101R: CTGTCAGAACAAGGTCGATGTTG) were designed with Primer Express^®^ Software v3.0.1 (Thermo Fisher Scientific, Waltham—Massachusetts, USA). The primers’ efficiency and correlation coefficient were E = 102.8% and R^2^ = 0.991, respectively.

### Methylation status and DNA sequence comparison

Genomic DNA was extracted in three biological replicates from young leaves of F_7_ individuals and the parental cultivars using the Invisorb Spin Mini Plant Kit (Stratec Molecular, Berlin, Germany) according to the manufacturer´s instructions. To determine the methylation status, the DNA was bisulfite converted using the EZ DNA Methylation-Gold^TM^ Kit (Zymo Research, Irvine—California, USA). The amplification of the converted DNA was carried out using conditions and primers for the “Region II” as described by Sun et al. [18]. This region is associated with significant differences in the methylation of CpG islands between photoperiod-insensitive and photoperiod-sensitive varieties. The primers spanned the region from -1,250 to -778 bp from the start codon. Since the primers used by Sun et al. [18] for the amplification of the unconverted genomic DNA did not perform reliably in our materials, new primers were designed (PromF2: GCCTTACGCACATCATCAGC and PromR2: GGTGACGTGGACGAAATGGA) that spanned the region from -1,220 to -659 bp. The primer specificity was verified using DNA amplified from chromosomes 2A, 2B and 2D of both parental cultivars as the template for the PCR. The chromosomes were purified by flow cytometric sorting after labelling GAA microsatellites using FISHIS [29], and their DNA was amplified according to Šimková et al. [30].

PCR was performed at 95°C for 13 min for the initial denaturation, followed by 34 cycles of 95°C for 30 s, 60°C for 30 s and 72°C for 1 min, and a final step at 72°C for 5 min. All amplifications were performed using the C1000 Touch TM Thermal cycler (Bio-Rad, Hercules—California, USA).

The PCR amplicons from both the original genomic DNA and the bisulfite converted genomic DNA of the parental cultivars and F_7_ lines were purified using Agenocourt AMPure XP (Beckman Coulter, Brea—California, USA) and cloned using TOPO^®^ TA Cloning^®^ Kit for Sequencing (Invitrogen, Carlsbad—California, USA) according to the manufacturer’s instructions. Inserts from the DNA clones were amplified in a 20 μl reaction mixture comprising a single bacterial colony as the template. The reaction conditions were as follows: initial denaturation at 95°C for 13 min, followed by 35 cycles of 95°C for 35 s, 55°C for 35 s, and 72°C for 50 s with a final extension for 10 min at 72°C. The PCR products were purified and sequenced as previously described [28]. The sequences were trimmed and aligned using Geneious 5.6.4 (http://www.geneious.com, [26]). The methylation status was determined with the Kismeth online tool, which is available at http://katahdin.mssm.edu/kismeth/revpage.pl.

### Copy number assessment and SNP marker development

The *Ppd-B1* gene copy number in the F_2_ population and both parents was determined by iDNA Genetics (Norwich, UK) using the Taqman^®^ assay as previously described by Díaz et al. [3]. To assess the CNV in the F_7_ plants, a method based on SNP polymorphisms was used. The size of the *Ppd-B1* locus and a higher inter-copy identity prevents the sequencing of individual *Ppd-B1* copies from a single chromosome. Therefore, the sequence of *Ppd-B1* gene of the cultivar Sonora 64 (JF946486.1), which has three copies of the gene similarly to KF, was used to develop the markers. We identified four SNPs between Sonora 64 and P (DQ885762). A primer pair (GACTCCTGCCATGAGTTTTGATG and ACCGCAGTGTGACTTCGATTATC) was used to identify the Sonora 64-like allele and designed to overlap the SNPs located at positions -10,427 bp (G-A) and -10,656 bp (A-G) from the START codon. The marker was named *owm1001*. The PCR amplification was performed in a 15 μl reaction mixture under the following cycling conditions: initial denaturation at 95°C for 13 min, followed by 35 cycles of 95°C for 35 s, 55°C for 35 s and 72°C for 50 s with a final elongation of 10 min at 72°C. The amplicons were purified and sequenced as previously described by Ivaničová et al. [28]. The sequences were trimmed and aligned using the software Geneious 5.6.4 [26].

## Results

### Mapping and QTL analysis

**Fig 1:**
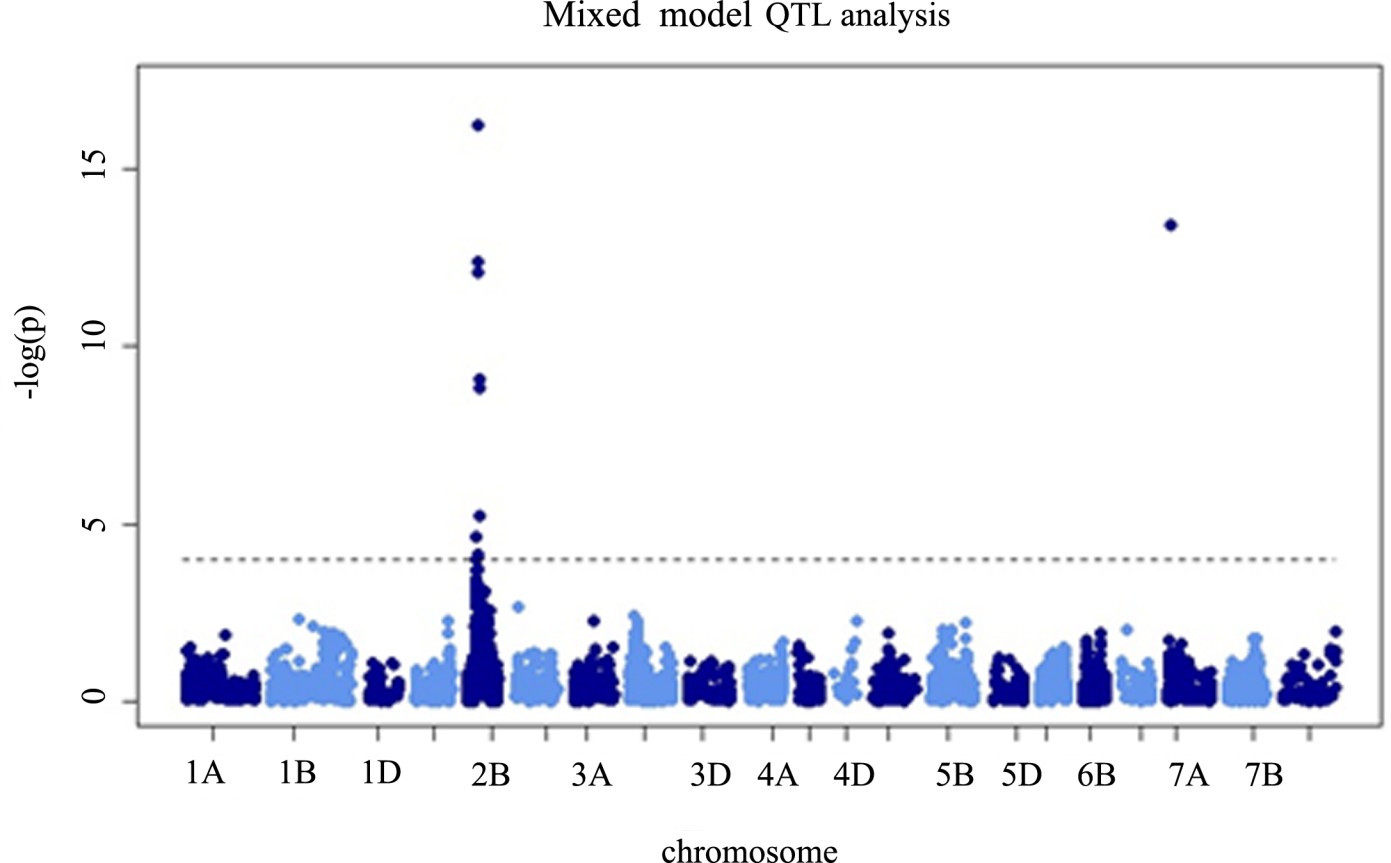
Whole genome QTL analysis of the heading time variation. Ninety F_2_ individuals were genotyped using the DArT markers. A mixed model QTL analysis revealed only one significant peak on short arm of chromosome 2B (2BS).

Ninety individuals of the F_2_ mapping population (30 from each of the early, intermediate and late flowering lines) were genotyped using the DArT technology [23]. In total, 5,069 DArT markers were found to be polymorphic and used to construct the genetic map. The heading times of the F_2_ lines ranged from 50 to 78 days. The QTL analysis revealed only one significant peak (LOD 15.6) on chromosome 2BS (Fig 1). Marker *owm1001*, which was derived from the *Ppd-B1* promoter region, mapped onto the peak, thus confirming that the heading time difference was mediated by the *Ppd-B1* gene. There was also one marker located on chromosome 7A (Fig 1), but further analyses showed that it was not significant.

### *VRN-A3* gene and *Ppd-B1* promoter sequence variation

The sequence of the *VRN-A3* gene (839 bp) and the adjacent 5´ end (1,750 bp) and 3´ end (143 bp) sequences were obtained from both parental cultivars. No sequence variations were identified. By comparing to the reference sequence of the cultivar Chinese Spring *FT* (*VRN-A3*) gene (EF428115), only one SNP (T → C) was shown, which was located 506 bp from the START codon in the first intron. This comparison confirmed that the putative marker association identified by the QTL analysis on chromosome 7A was most likely a bias.

A comparison of the sequence promoter and a portion of the first exon of the *Ppd-B1* gene (from—1,545 bp to + 251 bp) in the F_7_ lines 32_2 and 37_4 and the parental cultivars showed no variations.

### Copy number determination

Using the Taqman^®^ assay as previously described by Díaz et al. [3], we found that the early flowering cultivar KF carried three copies of *Ppd-B1* (designated the *Ppd-B1a* allele), while the late flowering cultivar P carried only one copy (designated the *Ppd-B1c* allele) per haploid genome. The same result was observed in the F_2_ individuals as follows: 30 early heading plants carried three copies, and 30 intermediate plants that are heterozygous in the *Ppd-B1* locus. Surprisingly, not all late plants had one copy as expected. Of these 30 late heading plants, 28 carried one copy of *Ppd-B1*, but two plants had three copies.

### Heading time

The mean heading time for KF was 50.6 ± 1.2 days, while the mean heading time for P was 86.2 ± 1.5 days. The heading time variation between the parents was observed under both SD and LD conditions. Offspring (generations F_2_—F_7_) derived from crossing the two cultivars were grown under LD conditions only to shorten the time to harvest the seeds and accelerate the attainment of the F_7_ generation with a homogenous background. The heading time of the F_2_ lines varied between 50–78 days and corresponded with the respective *Ppd-B1* CNV for a majority of plants (88 out of 90), i.e., plants with one copy of *Ppd-B1* headed later than plants with three copies. Surprisingly, we also observed heading time variations among the F_2_ lines with three copies of the gene. These lines (32_2 and 37_4) headed significantly later (17 days on average) than the early flowering parent KF with three gene copies. This phenomenon was observed repeatedly across the generations; however, a heading time analysis was performed only for the F_6_ and F_7_ generations. In the F_6_ generation (field conditions, natural LD), the mean heading times of lines 32_2 and 37_4 were 90.8 and 90.5 days, respectively, while the early parent KF headed in 78 days, and the late parent P headed in 96.1 days. These late lines and one early line (11_6, serving as a control) carried three *Ppd-B1* copies and were, therefore, selected for further analyses in the F_7_ generation.

A phenotypic analysis of the F_7_ lines (controlled regime, artificial LD) confirmed the results obtained in the F_2_ mapping population. The early line 11_6 headed at 52.2 ± 2.2 days on average, while the late lines 37_4 and 32_2 headed at 66.6 ± 4.7 and 74.1 ± 5.2 days on average, respectively. As previously mentioned, all lines (both early and late) had the *Ppd-B1a* allele (three copies). The early parent KF carries the same allele, and thus, late flowering was unexpected in these two lines. To clarify this observation, we assessed the *Ppd-B1* expression level and methylation status of the lines to determine the reason for the large differences in the heading times (16–24 days) between the lines with the same *Ppd-B1* copy number. Prior to the expression analysis, the lines were genotyped with markers (*Vrn-A1a*, *Vrn-B1c*, *vrn-D1*, *vrn-B3*, *Ppd-D1b* and *Ppd-A1b*) to exclude the possibility of outcrossing during the development of the F_7_ generation.

### *Ppd-B1* expression

**Fig 2:**
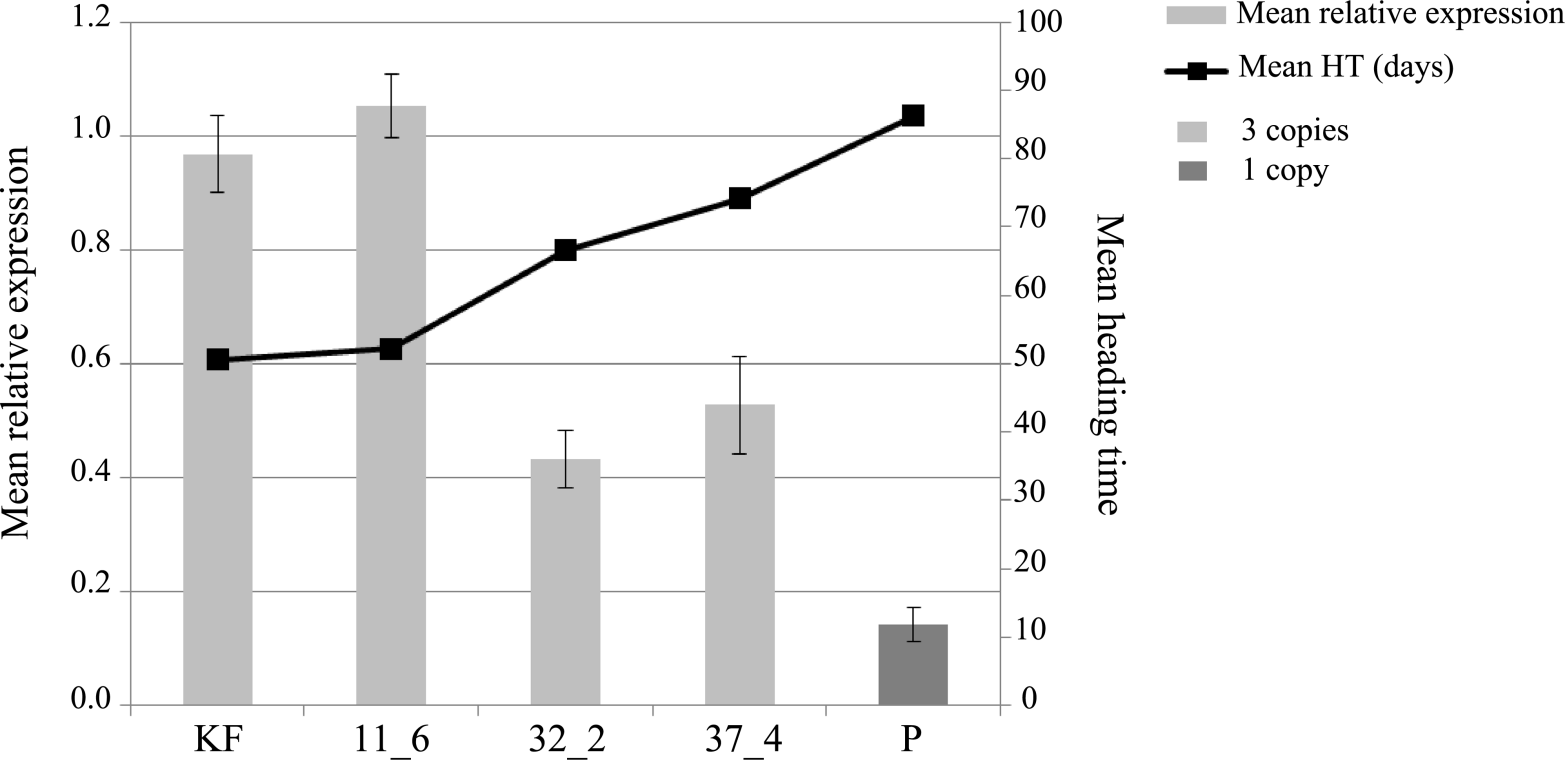
Mean relative expression of the *Ppd-B1* allele and the mean heading times of the parental cultivars and the F_7_ lines. 11_6—early line; 32_2 and 37_4—late lines; KF—early parental cultivar Kaerntner Frueher; P—late parental cultivar Paragon; HT—heading time. The data were derived from three biological replicates. The mean standard error of the relative expression is shown for each line.

The *Ppd-B1* expression level (Fig 2) was determined using a RT-qPCR assay with *Ppd-B1* specific primers. As expected, plants in the early flowering line had expression levels that were similar to those of KF, while the plants in the late flowering lines had a decreased expression level.

The differences between the late lines 32_2 and 37_4 and late parent P were statistically significant at the P<0.05 level. The expression levels in the early line 11_6 and early parent KF were not significantly different, while the differences between the early line 11_6 and late lines 32_2 and 37_4 were also significant at the P<0.05 level. The lower expression of *Ppd-B1* in P correlated with the delayed heading time (r = -0.955, P = 0.00001). However, the gene expression levels in lines 32_2 and 37_4 did not correlate with the respective *Ppd-B1* copy number.

### Methylation status of the *Ppd-B1* promoter region

**Fig 3:**
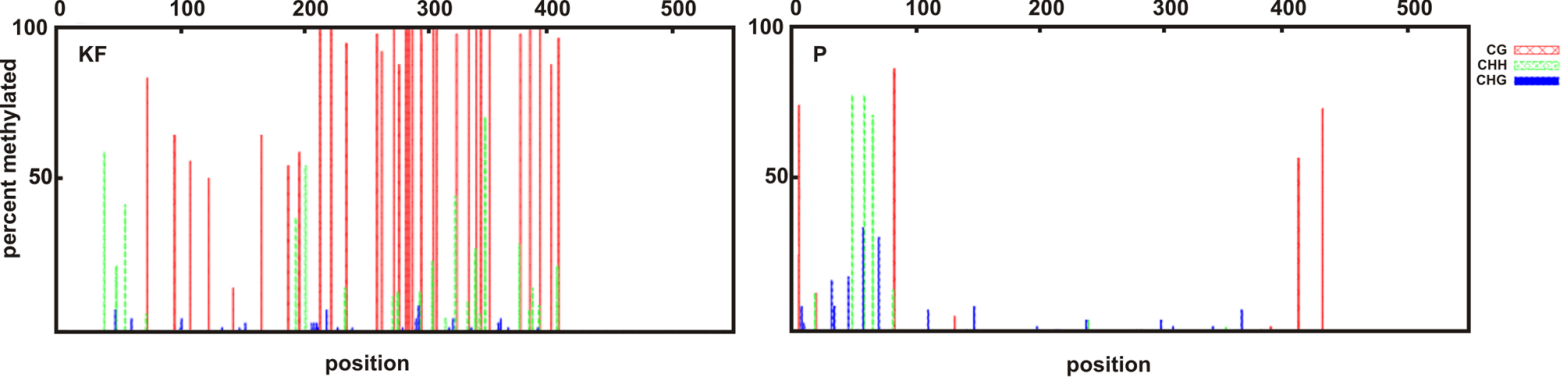
Comparison of the methylation status of the parental cultivars. The methylation level in the promoter region (442 bp) of the *Ppd-B1* gene from parental cultivars KF and P was analysed. The x-axis shows the cytosine positions in the analysed region, and the y-axis shows the percent of methylated CpG islands. A major difference was observed in the CG sites, and cultivar P had an 8.8-fold lower methylation level. Red lines represent the methylation of CG sites, the green lines represent the CHG sites and the blue lines represent the CHH sites.

The amplification of the unconverted genomic DNA with the newly designed primers resulted in an amplicon size of 561 bp, while the amplicon size of Region II using the primers designed by Sun et al. [18] was 472 bp. The combination of the sequencing data from the two amplicons resulted in a common 442 bp region that was used to assess the methylation status. The sequencing of at least 70 subclones per line revealed that the early parental variety KF with three copies of *Ppd-B1* had 85.69% of CG methylated, while the late P variety with one copy had only 9.97% of CG methylated (Table 2, Fig 3). The early line 11_6 showed a methylation status that was similar to that of KF (mean value 90.22%, Table 2, S1 Fig). Late line 32_2 had a slightly decreased methylation level of CG (mean value 70.61%, Table 2, S1 Fig), and this difference was statistically significant (P<0.01). Late line 37_4 showed a repeatedly high level of methylation (mean value 91.73%, Table 2, S1 Fig), which did not correspond with either the late heading or the lower expression level of *Ppd-B1*. Therefore, we have sequenced the promoter regions of the late lines 37_4 and 32_2 and parental cultivar KF. No polymorphisms were identified by comparing the promoter region sequences with parental cultivar P.

**Table 2:** Methylation status of the CpG islands in the early and late flowering lines (F_7_ generation).

Line	Plant	Methylation status (%)				*Ppd-B1* copies
		CG	Mean CG with SEM	CHG	CHH	
Early parent KF	121	85.69	87.17 ± 0.76	14.69	1.6	3
	123	87.62		12.37	2.69	
	129	88.21		13.24	2.09	
11_6	7	88.62	90.22 ± 1.02	16.64	0.70	3
	12	92.12		19.77	1.22	
	19	89.92		15.21	1.13	
32_2	101	74.08	70.61 ± 1.79	17.26	1.95	3
	104	69.66		11.82	1.14	
	109	68.10		9.81	1.13	
37_4	111	92.73	91.73 ± 0.79	19.48	1.65	3
	112	90.16		16.99	1.62	
	117	92.30		15.47	1.09	
Late parent P	134	11.6	9.97 ± 0.86	11.11	2.35	1
	135	8.68		12.31	1.68	
	136	9.64		14.54	1.95	

11_6—early line; 32_2 and 37_4—late lines; KF—early parental cultivar Kaerntner Frueher; P—late parental cultivar Paragon; SEM—standard error of mean

## Discussion

### Heading time and *Ppd-B1* CNV

The difference in the heading time between parental cultivars KF and P was first detected in 2010 during experiments focused on heading date variations due to a novel *Vrn-B1* allele [22]. The difference ranged from 20 to 40 days depending on the growth conditions (LD or SD) and was reproducible in several replications in both field and growth room trials. Surprisingly, both varieties have the same set of the following major flowering time alleles: *Vrn-A1*, *Vrn-B1c*, *vrn-D1*, *Vrn-A3*, *vrn-B3*, *Ppd-A1b* and *Ppd-D1b*. Even with identical major flowering time genes (vernalization and photoperiod) or saturation of their requirements, variations in the flowering time could still be observed. Such changes or variations are usually very subtle and represent a fine-tuning of the flowering time by so-called earliness *per se* genes. Earliness *per se* genes usually fine-tune the flowering time based on environmental conditions that are different from the vernalization and photoperiod. The earliness *per se* genes do not contribute to the flowering time variations dramatically, but under certain conditions, their contribution could be significant. The *Eps-A1* gene from chromosome 1A^m^ of *T*. *monococcum* is a good example of such a gene [31,32]. This gene under field conditions contributes to the flowering time variation by four to six days, but under fluorescent light and a temperature of 16°C or 23°C, the flowering time difference could be 80 and 50 days, respectively, [31]. This gene has a pleiotropic effect and affects all developmental phases from the double ridge stage to the heading stage [33].

In the present work, the QTL analysis of the F_2_ mapping population identified a single significant peak (LOD 15.6) on chromosome arm 2BS at the position of the *Ppd-B1* gene. Over 5,000 DArT markers were used in the QTL analysis, which provided enough sensitivity to exclude the effect of other loci, including the earliness *per se* genes. However, by assessing the CNV of the *Ppd-B1* alleles [3], we confirmed that the early flowering cultivar KF carried three copies of *Ppd-B1* (designated the *Ppd-B1a* allele), while the late flowering cultivar P carried one copy only (designated the *Ppd-B1c* allele) per haploid genome.

Similarly, Díaz et al. [3] and Cane et al. [34] observed variations in the heading time due to differences in the copy number of the *Ppd-B1* gene. Earlier flowering corresponded to a higher level of *Ppd-B1* gene expression in the lines with the three gene copies than in those with only one copy of the *Ppd-B1* gene; thus, a shorter time was necessary for the plants to achieve a critical amount of the Ppd-B1 protein and an earlier initiation of flowering. In our work, we observed a significant heading time variation in the F_2_ mapping population (derived from crossing the cultivars KF and P) with the same number of *Ppd-B1* gene copies. Cane et al. [34] observed a similar effect—plants with two copies had a longer heading time than plants carrying only one copy. The authors speculated that they identified a different *Ppd-B1* allele than the allele studied by Díaz et al. [3]. According to Díaz et al. [3], the cultivar Chinese Spring with three intact and one truncated *Ppd-B1* copies had a longer flowering time than plants with two copies of the gene. The variability in the heading time in the lines with the same copy number of flowering time gene (*HvFT1*) was also detected in barley [13]. Nitcher et al. observed that the heading time in F_2_ individuals of BGS213 × IMC mapping population with five copies of the *HvFT1* gene ranged from 30 to 39 days, while plants with three copies headed between 40 and 79 days, and plants with one copy headed between 100 and 125 days.

The effect of the CNV of the *Ppd-B1* gene on the heading date was studied recently by Würschum et al. [35], who showed the importance of the CNV for the heading time and wheat adaptation to different environmental conditions. The authors screened a panel of 1,100 winter wheat varieties to determine the frequency and geographic distribution of the CNVs at the *Ppd-B1* and *Vrn-A1* loci along with their effect on the flowering time. Their study confirmed that an increase in the number of *Ppd-B1* gene copies reduced the number of days required for heading. Interestingly, in contrast to the general trend, varieties from the Balkan region flowered later and had higher *Ppd-B1* copy numbers. We observed the same effect in the line with the fixed genomes that were isogenic for the major flowering time genes. Thus, we speculated that the difference in the flowering time in the line with the same *Ppd-B1* copy number was due to an altered *Ppd-B1* gene expression. To validate this hypothesis, we used the F_7_ generation of the mapping population for further analyses.

### *Ppd-B1* expression and promoter methylation

The expression of *Ppd-B1* increases quite rapidly, peaks three hours after dawn, and then decreases and oscillates close to the level maintained during the dark period [3]. This phenomenon can be observed in photoperiod-sensitive and photoperiod-insensitive plants. However, the difference in the gene expression between both types of plants is relatively small. We analysed the *Ppd-B1* expression in both parents and in early and late lines with three *Ppd-B1* copies and found a robust correlation between the heading time and the expression level (P = 0.00001). As expected, the late cultivar P had a lower relative expression than the early cultivar KF. The early line 11_6 with three copies had the same expression as KF, while the late lines (32_2 and 37_4) had a decreased expression (P<0.05) that was still higher than that of the late parental cultivar P (Fig 2).

The increase in the copy number of the *Ppd-B1* gene and the higher methylation level in its promoter appear to be associated with a higher expression, which, in turn, is associated with an acceleration of flowering and even a loss of photoperiod sensitivity. Sun et al. [18] reported a link between the methylation of the *Ppd-B1* allele, increased CNV and a higher level of *Ppd-B1* expression. Wheat cultivars with three copies of the *Ppd-B1* gene have more methylated CpG islands than varieties with one gene copy [18]. The authors described the following six regions in the *Ppd-B1* gene: regions I—IV are located in the promoter (upstream the coding region) and regions V and VI are located in the coding region, spanning exons 1–3. The most significant difference in the CpG methylation between the photoperiod-insensitive and photoperiod-sensitive cultivars was observed in Region II. The authors characterized the “*a”* and “*b”* methylation haplotypes as follows: the *a* haplotype (higher methylation) was associated with a higher gene copy number and exhibited a higher expression of *Ppd-B1* than the varieties with the “*b”* haplotype (lower methylation) and lower CNV. Nevertheless, several cultivars with a low methylation level and one *Ppd-B1* gene copy had a higher expression than cultivars with three or even four copies of the gene and a high methylation status. Although DNA methylation is usually connected with gene silencing [36], in the case of *Ppd-B1*, it appears to act in the opposite way. Sun et al. [18] also noted that methylated *Ppd-B1* promoter regions cover the same area, which is deleted in the *Ppd-A1a* and *Ppd-D1a* alleles; these alleles have a higher expression than their respective alleles without the deletion. The authors concluded that a deletion or methylation in this region may block putative repressor function and, therefore, lead to an enhanced *Ppd-B1* expression.

### Identity of copies and suggestive effect of paramutation

A general understanding of the “gene copy number” implies that extra copies are the same as the original, which may be true shortly after a duplication event. However, the individual gene copies may gradually accumulate differences, which may change gene expression or even function [37]. In addition, an increased copy number may compromise the stability of homeostasis, and the extra gene copies may be downregulated unless they present an evolutionary advantage.

The variation in the heading time between the wheat lines with different numbers of *Ppd-B1* gene copies may occur due to differences in the DNA sequences between the individual gene copies. Such differences were reported in the cultivar Chinese Spring, which has four copies of the *Ppd-B1* gene distributed over a 100 kb region, with truncation of the first copy [3]. As previously mentioned, Cane et al. [34] identified the *Ppd-B1* allele with two copies, but in contrast to the observations by Díaz et al.[34], this allele caused a longer flowering time. Cane et al. [34] explained the discrepancy by the possible existence of two different alleles. Würschum et al. [35] also suggested that individual copies of the *Ppd-B1* gene may differ in DNA sequences and are non-functional or have different functional properties. In fact, indel-related variation was found to have a more significant effect on the flowering time than the variation in the copy number [38].

To clarify this issue, we sequenced a portion of the promoter region and *Ppd-B1a* allele from both parental lines, and no differences in the DNA sequence were found. This result implied that the effect on the flowering time was largely due to the differences in the copy number. A lower level of DNA methylation corresponds to lower gene expression and, subsequently, the later flowering time observed in line 32_2 with three copies of *Ppd-B1* than that of the early flowering cultivar KF with the same number of *Ppd-B1* copies. However, line 37–4, which also has three copies of *Ppd-B1*, had a higher methylation level than the early flowering cultivar KF and also flowered even later than line 32_2 (Table 2). This observation suggests that the optimal methylation status of the promoter activation would be close to values observed in the early cultivar KF (87%) and line 11_6 (90%) (Table 2). In this case, approximately 71% (line 32_2) may not be enough for promoter activation, and the 92% (line 37_4) may have an inhibitory effect on expression.

The delay in the heading time detected in the plants with three copies of the *Ppd-B1* gene might be explained by a paramutation effect. Paramutation is an allelic interaction in which one allele, which is referred to as paramutagenic (in our study *Ppd-B1b*), causes a heritable change in the expression of a homoeologous paramutable (in our study *Ppd-B1a*) allele [39]. One can speculate that the decreased level of DNA methylation observed in line 32_2 (three copies) might be due to paramutation. Different epialleles may be responsible for the difference in the gene expression patterns and the following variable range of phenotypes (reviewed in [40]). The irregularities we identified in the heading times may be explained by the weak stability of epigenetic modifications at the DNA level [41].

In conclusion, we have described late flowering wheat lines carrying the same number of *Ppd-B1* copies (three) as the early flowering parental variety. A late flowering time was associated with a lower *Ppd-B1* expression than that in a parent with the same gene copy number. While the molecular mechanisms underlying this interesting phenomenon remain unclear, our results indicate that the CNV may not be responsible for complete gene penetrance. The CNV of the *Ppd-B1* allele has a clear impact on the heading date and, thus, should be employed as a source of variability in breeding programmes. The insensitive allele of *Ppd-B1* was identified recently, and thus, its mode of function is not well understood. Further investigations are needed to fully explain the behaviour of the *Ppd-B1* alleles and their effect on flowering time pathways.

## Supporting information

S1 Fig Comparison of the methylation status of the F_7_ lines. The methylation level in the promoter region (442 bp) of the *Ppd-B1* gene from F_7_ lines was analysed. The x-axis shows the cytosine positions in the analysed region, and the y-axis shows the percent of methylated CpG islands. Red lines represent the methylation of CG sites, the green lines represent the CHG sites and the blue lines represent the CHH sites.A-C: early line 11_6; D-F: late line 32_2; G-I: late line 37_4.
(PDF)

## References

[1] Beales J, Turner A, Griffiths S, Snape J, Laurie D. A pseudo-response regulator is misexpressed in the photoperiod insensitive Ppd-D1a mutant of wheat (Triticum aestivum L.). Theor Appl Genet. 2007;115: 721–733. doi: 10.1007/s00122-007-0603-4 17634915 10.1007/s00122-007-0603-4

[2] Nishida H, Yoshida T, Kawakami K, Fujita M, Long B, Akashi Y, et al Structural variation in the 5′ upstream region of photoperiod-insensitive alleles Ppd-A1a and Ppd-B1a identified in hexaploid wheat (Triticum aestivum L.), and their effect on heading time. Mol Breed. 2012;31: 27–37. doi: 10.1007/s11032-012-9765-0

[3] Díaz A, Zikhali M, Turner A, Isaac P, Laurie D. Copy number variation affecting the Photoperiod-B1 and Vernalization-A1 genes is associated with altered flowering time in wheat (Triticum aestivum). PLoS One. 2012;7: e33234 doi: 10.1371/journal.pone.0033234 22457747 10.1371/journal.pone.0033234 PMC3310869

[4] Zhang X, Gao M, Wang S, Chen F, Cui D. Allelic variation at the vernalization and photoperiod sensitivity loci in Chinese winter wheat cultivars (Triticum aestivum L.). Front Plant Sci. 2015;6: 1–10. doi: 10.3389/fpls.2015.00001 26191066 10.3389/fpls.2015.00470 PMC4486769

[5] Redon R, Ishikawa S, Fitch KR, Feuk L, Perry GH, Andrews TD, et al Global variation in copy number in the human genome. Nature. 2006;444: 444–54. doi: 10.1038/nature05329 17122850 10.1038/nature05329 PMC2669898

[6] Zmieńko A, Samelak A, Kozłowski P, Figlerowicz M. Copy number polymorphism in plant genomes. Theor Appl Genet. 2014;127: 1–18. doi: 10.1007/s00122-013-2177-7 23989647 10.1007/s00122-013-2177-7 PMC4544587

[7] Iafrate AJ, Feuk L, Rivera MN, Listewnik ML, Donahoe PK, Qi Y, et al Detection of large-scale variation in the human genome. Nat Genet. 2004;36: 949–951. doi: 10.1038/ng1416 15286789 10.1038/ng1416

[8] Conrad DF, Pinto D, Redon R, Feuk L, Gokcumen O, Zhang Y, et al Origins and functional impact of copy number variation in the human genome. Nature. 2012;464: 704–712. doi: 10.1038/nature08516.Origins 10.1038/nature08516 PMC3330748 19812545

[9] Singleton AB, Farrer M, Johnson J, Singleton AB, Hague S, Kachergus J, et al -Synuclein Locus Triplication Causes Parkinson’s Disease. Science (80-). 2003;302: 841–841. doi: 10.1126/science.1090278 14593171 10.1126/science.1090278

[10] Dumas M, Sadick N, Noblesse E, Juan M, Lachman-Weber N, Boury-Jamot M, et al Hydrating skin by stimulating biosyntheis of aquaporins. J Drugs Dermatology. 2008;6(6 Suppl): 20–24. 17691206

[11] Saintenac C, Jiang D, Akhunov ED. Targeted analysis of nucleotide and copy number variation by exon capture in allotetraploid wheat genome. Genome Biol. BioMed Central Ltd; 2011;12: R88 doi: 10.1186/gb-2011-12-9-r88 21917144 10.1186/gb-2011-12-9-r88 PMC3308051

[12] Francia E, Morcia C, Pasquariello M, Mazzamurro V, Milc JA, Rizza F, et al Copy number variation at the HvCBF4–HvCBF2 genomic segment is a major component of frost resistance in barley. Plant Mol Biol. Springer Netherlands; 2016; 161–175. doi: 10.1007/s11103-016-0505-4 27338258 10.1007/s11103-016-0505-4

[13] Nitcher R, Distelfeld A, Tan C, Yan L, Dubcovsky J. Increased copy number at the HvFT1 locus is associated with accelerated flowering time in barley. Mol Genet Genomics. 2013;288: 261–75. doi: 10.1007/s00438-013-0746-8 23591592 10.1007/s00438-013-0746-8 PMC3664738

[14] Ehrlich M, Gama-Sosa M, Huang L, Midgett R, Kenneth C, Mccune R, et al Amount and distribution of 5-methylcytosine in human DNA from different types of tissues of cells. Nucleic Acid Res. 1982;10: 2709–2721. 7079182 10.1093/nar/10.8.2709 PMC320645

[15] Henderson IIRI, Chan SSRS, Cao X, Johnson L, Jacobsen SSESSE. Accurate sodium bisulfite sequencing in plants. Epigenetics. 2010;5: 47–49. doi: 10.4161/epi.5.1.10560 20081358 10.4161/epi.5.1.10560 PMC2829377

[16] Bird A. The essentials of DNA methylation. Cell. 1992;70: 5–8. doi: 10.1016/0092-8674(92)90526-I 1377983 10.1016/0092-8674(92)90526-i

[17] Keshet I, Lieman-Hurwitz J, Cedar H. DNA methylation affects the formation of active chromatin. Cell. 1986;44: 535–543. doi: 10.1016/0092-8674(86)90263-1 3456276 10.1016/0092-8674(86)90263-1

[18] Sun H, Guo Z, Gao L, Zhao G, Zhang W, Zhou R, et al DNA methylation pattern of Photoperiod-B1 is associated with photoperiod insensitivity in wheat (Triticum aestivum). New Phytol. 2014; 1–11. doi: 10.1111/nph.12948 25078249 10.1111/nph.12948

[19] Diallo A, Ali-Benali M, Badawi M, Houde M, Sarhan F. Expression of vernalization responsive genes in wheat is associated with histone H3 trimethylation. Mol Genet Genomics. 2012;287: 575–90. doi: 10.1007/s00438-012-0701-0 22684814 10.1007/s00438-012-0701-0

[20] Finnegan E, Dennis E. Vernalization-induced trimethylation of histone H3 lysine 27 at FLC is not maintained in mitotically quiescent cells. Curr Biol. 2007;17: 1978–83. doi: 10.1016/j.cub.2007.10.026 17980595 10.1016/j.cub.2007.10.026

[21] Sung S, Schmitz R, Amasino R. A PHD finger protein involved in both the vernalization and photoperiod pathways in Arabidopsis. Genes Dev. Cold Spring Harbor Laboratory Press; 2006;20: 3244–3248. doi: 10.1101/gad.1493306 17114575 10.1101/gad.1493306 PMC1686601

[22] Milec Z, Tomková L, Sumíková T, Pánková K. A new multiplex PCR test for the determination of Vrn-B1 alleles in bread wheat (Triticum aestivum L.). Mol Breed. 2012;30: 317–323. doi: 10.1007/s11032-011-9621-7

[23] Piepho HP. A mixed-model approach to mapping quantitative trait loci in Barley on the basis of multiple environment data. Genetics. 2000;156: 2043–2050. 11102394 10.1093/genetics/156.4.2043 PMC1461386

[24] Akbari M, Wenzl P, Caig V, Carling J, Xia L, Yang S, et al Diversity arrays technology (DArT) for high-throughput profiling of the hexaploid wheat genome. Theor Appl Genet. 2006;113: 1409–1420. doi: 10.1007/s00122-006-0365-4 17033786 10.1007/s00122-006-0365-4

[25] IWGSC, Mayer K. A chromosome-based draft sequence of the hexaploid bread wheat (Triticum aestivum) genome. Science. 2014;345: 1251788 doi: 10.1126/science.1251788 25035500 10.1126/science.1251788

[26] Kearse M, Moir R, Wilson A, Stones-Havas S, Cheung M, Sturrock S, et al Geneious Basic: An integrated and extendable desktop software platform for the organization and analysis of sequence data. Bioinformatics. 2012;28: 1647–1649. doi: 10.1093/bioinformatics/bts199 22543367 10.1093/bioinformatics/bts199 PMC3371832

[27] Ye J, Coulouris G, Zaretskaya I, Cutcutache I, Rozen S, Madden TL. Primer-BLAST: A tool to design target-specific primers for polymerase chain reaction. BMC Bioinformatics. 2012;13: 134 doi: 10.1186/1471-2105-13-134 22708584 10.1186/1471-2105-13-134 PMC3412702

[28] Ivanicova Z, Jakobson I, Reis D, Safar J, Milec Z, Abrouk M, et al Characterization of new allele influencing flowering time in bread wheat introgressed from Triticum militinae. N Biotechnol. 2016;33: 718–727. doi: 10.1016/j.nbt.2016.01.008 26899284 10.1016/j.nbt.2016.01.008

[29] Giorgi D, Farina A, Grosso V, Gennaro A, Ceoloni C, Lucretti S. FISHIS: Fluorescence In Situ Hybridization in Suspension and Chromosome Flow Sorting Made Easy. PLoS One. 2013;8 doi: 10.1371/journal.pone.0057994 23469124 10.1371/journal.pone.0057994 PMC3585268

[30] Šimková H, Svensson JT, Condamine P, Hribová E, Suchánková P, Bhat PR, et al Coupling amplified DNA from flow-sorted chromosomes to high-density SNP mapping in barley. BMC Genomics. 2008;9: 294 doi: 10.1186/1471-2164-9-294 18565235 10.1186/1471-2164-9-294 PMC2453526

[31] Bullrich L, Appendino M, Tranquilli G, Lewis S, Dubcovsky J. Mapping of a thermo-sensitive earliness per se gene on Triticum monococcum chromosome 1Am. Theor Appl Genet. 2002;105: 585–593. doi: 10.1007/s00122-002-0982-5 12582508 10.1007/s00122-002-0982-5

[32] Valárik M, Linkiewicz A, Dubcovsky J. A microcolinearity study at the earliness per se gene Eps-A(m)1 region reveals an ancient duplication that preceded the wheat-rice divergence. Theor Appl Genet. 2006;112: 945–957. doi: 10.1007/s00122-005-0198-6 16432738 10.1007/s00122-005-0198-6

[33] Lewis S, Faricelli ME, Appendino ML, Valárik M, Dubcovsky J. The chromosome region including the earliness per se locus Eps-Am1 affects the duration of early developmental phases and spikelet number in diploid wheat. J Exp Bot. 2008;59: 3595–607. doi: 10.1093/jxb/ern209 18836186 10.1093/jxb/ern209 PMC2561150

[34] Cane K, Eagles H, Laurie D, Trevaskis B, Vailance N, Eastwood R, et al Ppd—B1 and Ppd—D1 and their effects in southern Australian wheat. Crop Pasture Sci. 2013;64: 100–114.

[35] Würschum T, Boeven PHG, Langer SM, Longin CFH, Leiser WL. Multiply to conquer: Copy number variations at Ppd-B1 and Vrn-A1 facilitate global adaptation in wheat. BMC Genet. BMC Genetics; 2015;16: 96 doi: 10.1186/s12863-015-0258-0 26219856 10.1186/s12863-015-0258-0 PMC4518651

[36] Newell-Price J, Clark AJL, King P. DNA methylation and silencing of gene expression. Trends Endocrinol Metab. 2000;11: 142–148. doi: 10.1016/S1043-2760(00)00248-4 10754536 10.1016/s1043-2760(00)00248-4

[37] Force a, Force a, Lynch M, Lynch M, Postlethwait J, Postlethwait J. Preservation of duplicate genes by subfunctionalization. Am Zool. 1999;39: 0. 10101175

[38] Kiss T, Balla K, Veisz O, Láng L, Bedő Z, Griffiths S, et al Allele frequencies in the VRN-A1, VRN-B1 and VRN-D1 vernalization response and PPD-B1 and PPD-D1 photoperiod sensitivity genes, and their effects on heading in a diverse set of wheat cultivars (Triticum aestivum L.). Mol Breed. 2014;34: 297–310. doi: 10.1007/s11032-014-0034-2 25076837 10.1007/s11032-014-0034-2 PMC4092236

[39] Della Vedova C, Cone K. Paramutation: The Chromatin Connection. Plant Cell. 2004;16: 1358–1364. doi: 10.1105/tpc.160630 15178748 10.1105/tpc.160630 PMC490031

[40] Pilu R. Seminars in Cell & Developmental Biology Paramutation phenomena in plants. Semin Cell Dev Biol. Elsevier Ltd; 2015;44: 2–10. doi: 10.1016/j.semcdb.2015.08.015 26335267 10.1016/j.semcdb.2015.08.015

[41] Herman JJ, Spencer HG, Donohue K, Sultan SE. How stable “should” epigenetic modifications be? Insights from adaptive plasticity and bet hedging. Evolution (N Y). 2014;68: 632–643. doi: 10.1111/evo.12324 24274594 10.1111/evo.12324

