# Correction: Heritable heading time variation in wheat lines with the same number of *Ppd-B1* gene copies

**DOI:** 10.1371/journal.pone.0192989

**Published:** 2018-02-12

**Authors:** Zuzana Ivaničová, Miroslav Valárik, Kateřina Pánková, Martina Trávníčková, Jaroslav Doležel, Jan Šafář, Zbyněk Milec

In the Funding section, the second grant number from the Ministry of Agriculture of the Czech Republic is missing and should read: This work was supported by the Ministry of Education, Youth and Sports of the Czech Republic (award LO1204 from the National Program of Sustainability I), Czech Science Foundation (grant no. P501-12-G090) and supported by Ministry of Agriculture of the Czech Republic, Project No. MZE ČR RO0417 and Project No. QK1710302.
