# Supplementary material for: Heritable heading time variation in wheat lines with the same number of Ppd-B1 gene copies
Source: PLoS One. 2017 Aug 28;12(8):e0183745. doi: 10.1371/journal.pone.0183745 (PMC5573275; doi:10.1371/journal.pone.0183745)

A) plant no. 7, line 11_6

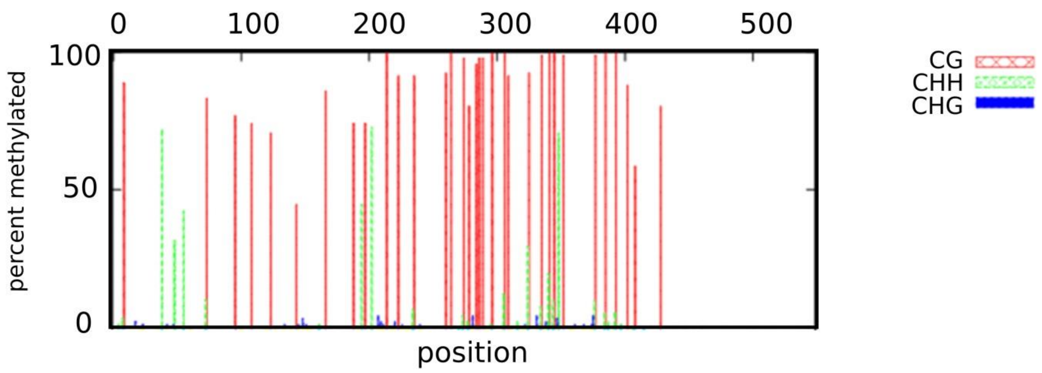

B) plant no. 12, line 11_6

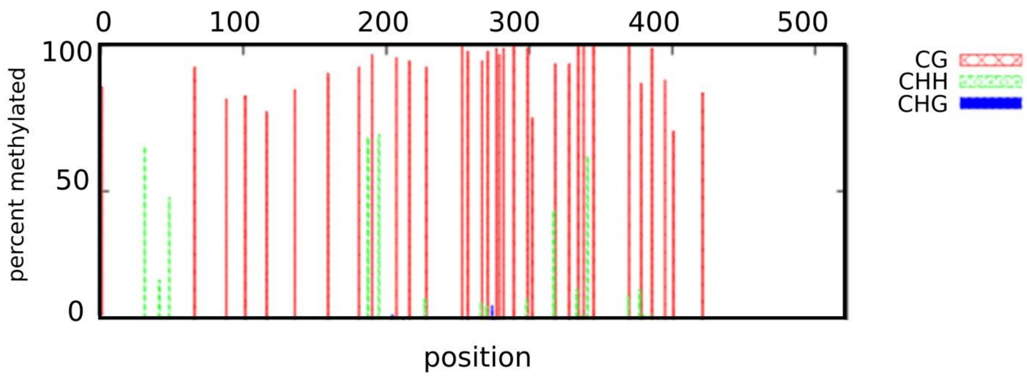

C) plant no. 19, 11_6

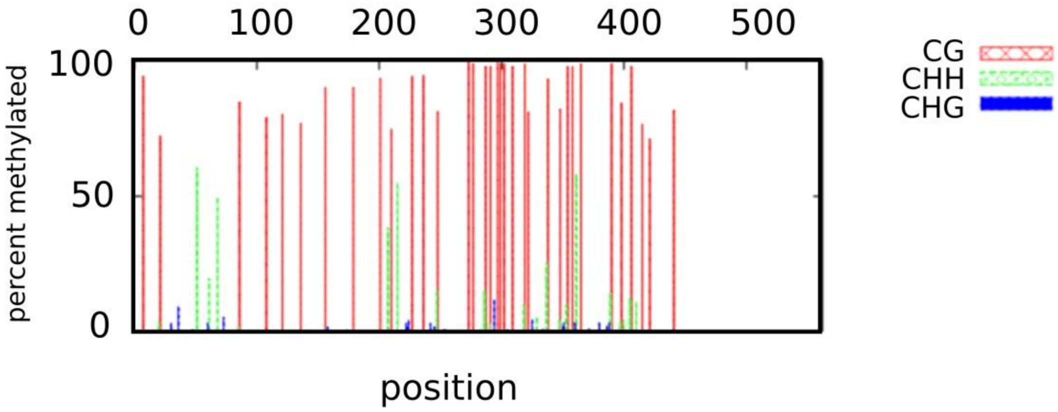

D) plant no. 101, line 32_2

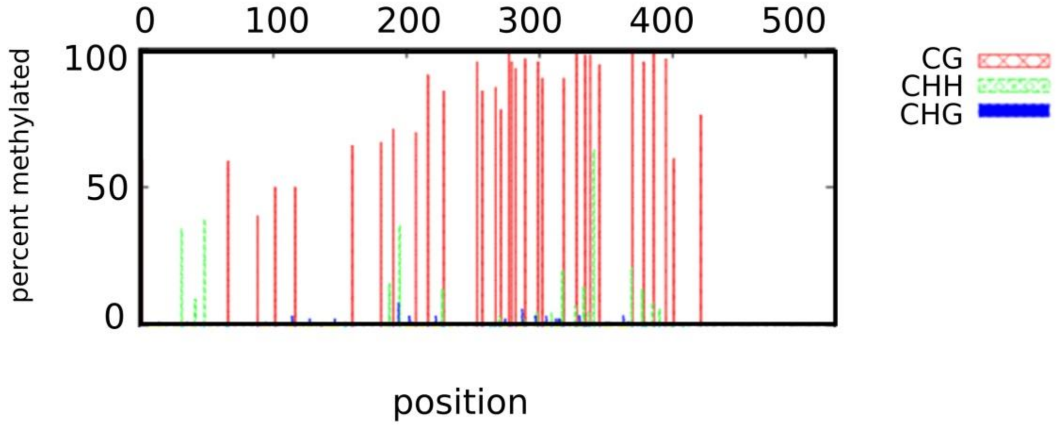

E) plant no. 104, line 32_2

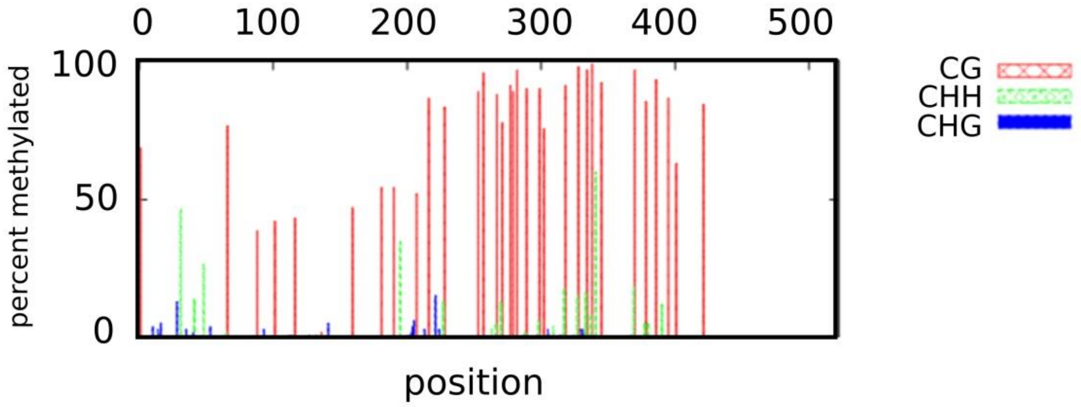

F) plant no. 109, line 32_2

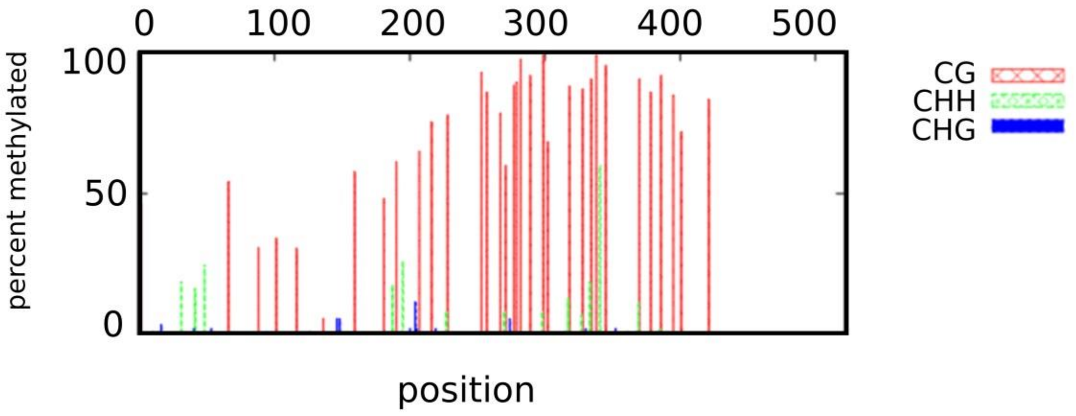

G) plant no. 111, line 37_4

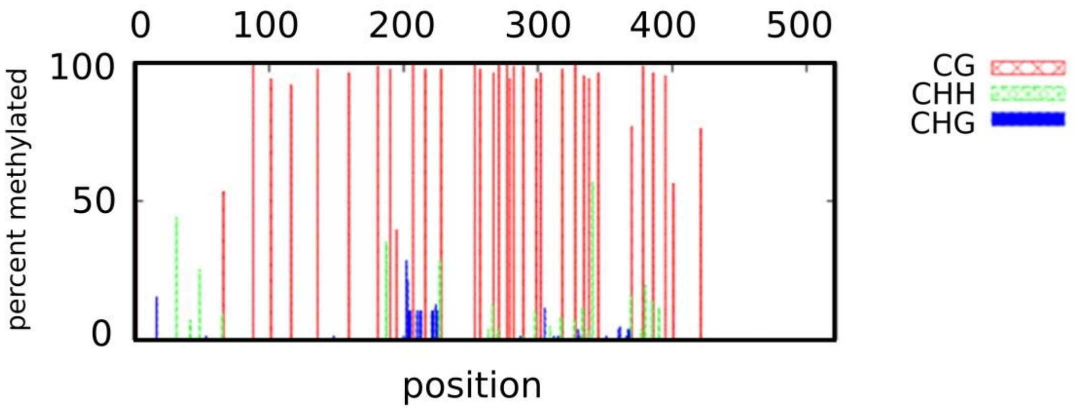

H) plant no. 112, line 37_4

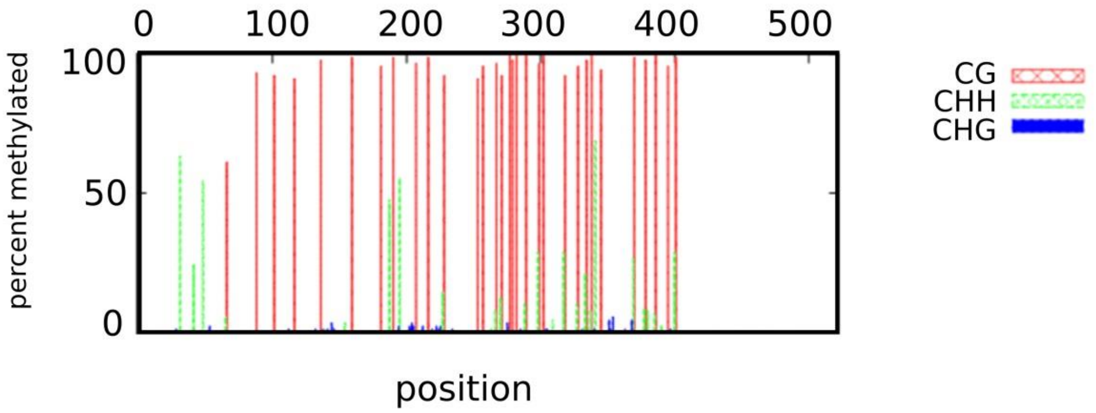

I) plant no. 117, line 37_4

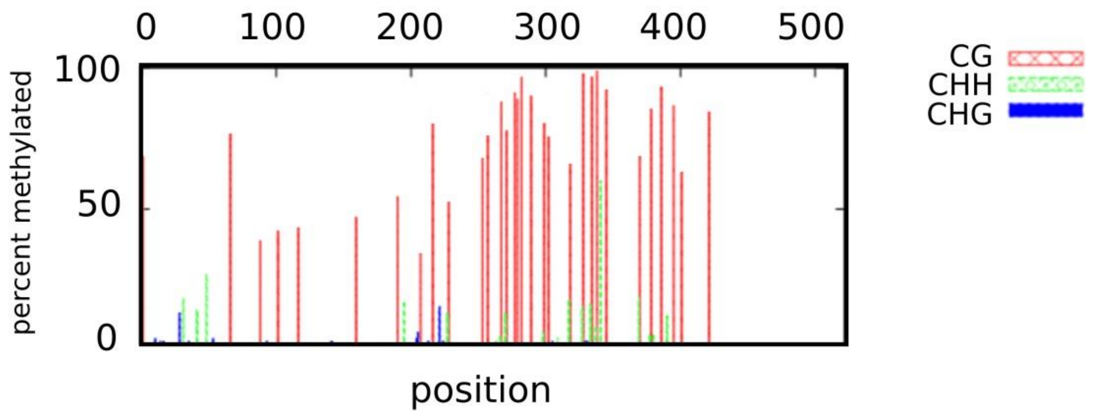

Supplement: S1 Fig — The methylation level in the promoter region (442 bp) of the Ppd-B1 gene from F7 lines was analysed. The x-axis shows the cytosine positions in the analysed region, and the y-axis shows the percent of methylated CpG islands. Red lines represent the methylation of CG sites, the green lines represent the CHG sites and the blue lines represent the CHH sites.A-C: early line 11_6; D-F: late line 32_2; G-I: late line 37_4. (PDF) [file pone.0183745.s001.pdf]
